# Platelet-rich plasma and ablative fractional carbon dioxide laser therapy for chronic scar management: a systematic review

**DOI:** 10.1007/s10103-026-04860-1

**Published:** 2026-04-22

**Authors:** Shonnelly Novintan, Daniel Campioni-Norman, Shina Ardani, Tara Mack, Quentin Frew

**Affiliations:** 1 St. Andrews Centre for Plastic Surgery and Burns, Mid-South Essex NHS Trust, Chelmsford, United Kingdom; 2 Imperial College London, London, United Kingdom; 3 Depart of Plastic Surgery, Southmead Hospital, Bristol, United Kingdom

**Keywords:** Ablative fractional carbon dioxide laser, Autologous platelet-rich plasma, Combined modality therapy, Scar, Burns

## Abstract

Purpose Scarring following acne, burns, and trauma remains a significant clinical and psychosocial challenge. Despite increasing clinical use, there is no consensus on the efficacy and safety of Ablative Fractional CO₂ Laser (AFCL) combined with Platelet-Rich Plasma (PRP) across scar aetiologies. This systematic review evaluated the efficacy and safety of AFCL with PRP for chronic acne, traumatic, and burn scars. Methods MEDLINE (PubMed) and Embase (Ovid) were searched to 1 September 2025 using terms related to scars, fractional CO₂ laser, and platelet-rich plasma. Eligible studies included human subjects treated with AFCL (10,600 nm) and autologous PRP delivered topically or intradermally. Studies using platelet-rich fibrin, PRP gel, or non-synchronous administration were excluded. Risk of bias was assessed using RoB 2 and ROBINS-I. Results Seventeen studies (*n* = 420) met inclusion criteria. Nine (53%) were randomised studies, six (29%) non-randomised, and two (12%) retrospective. AFCL settings ranged from 10 to 30 W, dwell times 0.1–1.54 ms, and spot spacing 0.5–2.0 mm. PRP was predominantly prepared via double-spin centrifugation and delivered topically (47%) or intradermally (59%). AFCL+PRP showed superior efficacy to AFCL alone, with significant improvements in scar texture, pigmentation, erythema, and depth. Patient satisfaction was consistently higher with combination therapy, and topical PRP notably reduced downtime, suggesting accelerated repair and modulated inflammation. Conclusions AFCL combined with PRP enhances scar remodelling and patient outcomes while reducing complications. Heterogeneity in laser parameters, PRP preparation, and outcome measures limits direct comparison. Robust multicentre randomised trials are needed to establish parameter-specific guidelines for AFCL-PRP therapy.

**Supplementary Information:**

The online version contains supplementary material available at 10.1007/s10103-026-04860-1.

## Introduction

Scarring is highly prevalent across a range of conditions, including acne, burns, and trauma. Among individuals with acne, around 47% develop scars, whereas burn injuries result in scarring in 32 to 72% of cases, with an annual admission rate of 0.29 per 1,000 individuals in the UK [1, 2]. The management of these conditions imposes a substantial financial burden on healthcare systems [3, 4]. Moreover, scarring has a profound impact on patients’ quality of life, contributing to physical symptoms such as pain, pruritus, and restricted mobility, as well as significant psychological distress with high prevalence of depression and post-traumatic stress disorder [5–8].

Residual scarring often requires further treatment to improve the outcome; functionally, aesthetically and psychologically. However, a significant and pervasive challenge relating to chronic scar management is the lack of standardised guidance on treatment methods and protocols, with no consensus as to the optimal treatment regime. National Institute for Health and Care Excellence (NICE, UK) does not provide guidelines for management of hypertrophic burns scars, whilst the National Burn Care Standards (2018) state that laser treatment should be provided as an option for burn scar patients [9]. Minimal national guidance is available in the UK for atrophic acne-related scarring (NG198) [10]. Specific treatment details are often dictated by anecdotal evidence and expert consensus [11].

Ablative fractional carbon dioxide laser (AFCL) therapy is increasingly seen as an efficacious and safe treatment for chronic scar management, with growing evidence within the literature [12, 13]. AFCL uses fractional photothermolysis, creating controlled microthermal zones that ablate the epidermis while preserving surrounding and deeper dermal tissue, to promote rapid re-epithelialisation. This localised photothermal effect vaporises epidermal layers and triggers dermal repair processes, stimulating fibroblast activation, collagen remodelling, and elastic fibre reorganisation, ultimately improving tissue structure and scar appearance [14].

Alongside this, platelet rich plasma (PRP), often seen as a staple within regenerative and rejuvenation focused dermal treatments, is seeing greater utility as an adjunctive therapy complementing AFCL. PRP is an autologous plasma concentrate, providing a reservoir of bioactive mediators for repair. Upon activation, platelets release alpha-granule growth factors, including PDGF TGF-β1/2, EGF, and VEGF, which drive cellular proliferation, differentiation, angiogenesis, and chemotaxis [15, 16]. Dense granules simultaneously release vasoactive molecules transiently increasing vascular permeability and modulating local immune responses [17]. This paracrine signalling recruits stem cells, enhances proliferation and lineage commitment, and tempers excessive inflammation, promoting efficient re-epithelialisation [18].

The potential utility and benefit of these treatments are being limited by insufficient and low-quality evidence, alongside a lack of standardised treatment guidelines. To address this problem, the current treatment practices and outcomes need to be quantified. Previous systematic reviews have investigated the combined role of AFCL alongside PRP in patients with acne scars, however, there has yet been a review that examines wider usage in all scar patients, which could yield important lessons that could be drawn and shared across patients with burn and traumatic scars. The primary aim of this study is to systematically review the use of PRP and AFCL therapy for chronic scar management.

## Methodology

### Rationale

The review was initially designed to evaluate AFCL combined with PRP specifically in burn scars. Preliminary searches identified only two eligible studies, which precluded a meaningful synthesis of outcomes. To generate a more comprehensive understanding of the therapeutic potential of AFCL + PRP, the inclusion criteria was broadened to encompass traumatic and acne scars. This approach was selected because acne scars represent the most extensively studied population for this combination therapy, allowing insights from these studies to inform future research and clinical protocol development in burn scar management.

### Search strategy

The systematic review was conducted according to the Preferred Reporting Items for Systematic Reviews and Meta-Analysis (PRISMA) guidelines. Embase (Ovid) and MEDLINE (PubMed) were searched systematically up to 1 st September 2025. The search strategy included three key search terms which were “scars”, “lasers” and “platelet-rich plasma” alongside synonyms and related subject headings, combined with the Boolean operators ‘AND’ and ‘OR’. Reference lists of included papers were screened for additional papers.

Two authors (SN, DCN) assessed all studies from the primary search independently for their relevance, initially reviewing titles and abstracts. This was carried out using systematic review software Covidence (Veritas Health Innovation, Australia). In case of disagreement regarding study inclusion a third reviewer (QF) was consulted. Case reports, conference abstracts and study protocols were excluded. Only studies published in English were included.

Studies were included reporting patients with acne, traumatic or burn scars, who received AFCL resurfacing with subsequent application of autologous PRP. Exclusion criteria included (i) patients who were pregnant, (ii) had a history of keloid scar or active inflammation, (iii)used platelet-rich fibrin, leukocyte-rich fibrin or PRP gel, (iv) intradermal PRP with added activators in the final preparation stage, (vi) additional treatments e.g. hyaluronic or fat grafting and vi) studies were excluded if AFCL and PRP were not administered concurrently, but instead delivered at separate time points.

### Data extraction

An extraction framework was established with the senior author (QF), and data were extracted by a single author (SN). Data were extracted in 4 domains: (i) methods; (ii) population- scar aetiology, demographics, classification; (iii) interventions – laser pulse energy, laser wavelength, laser power, laser passes, laser density, spot size, dwell time, spacing, PRP spin settings, PRP administration route, PRP preparation; (iv) outcomes – clinical improvement, scar assessment scales, patient satisfaction, adverse events, other digital assessment tools. In studies with comparator interventions data was only extracted from the AFCL-PRP subgroup.

### Quality assessment

Risk of bias in the included studies was evaluated using the RoB 2 tool [19] for randomised studies and the ROBINS-I tool [20] for non-randomised studies by authors (SN, SA). Discrepancies were resolved through discussion with the senior author (QF). Each study was categorised as having a low, moderate, serious or critical risk of bias (Supplementary Figs. 1 and 2).

Considerable clinical and methodological heterogeneity was present across studies, including differences in PRP preparation, AFCL parameters, and outcome assessment scales. Due to this variability, a meta-analysis was not performed, and findings were summarised narratively.

## Results

### Included studies

**Fig. 1:**
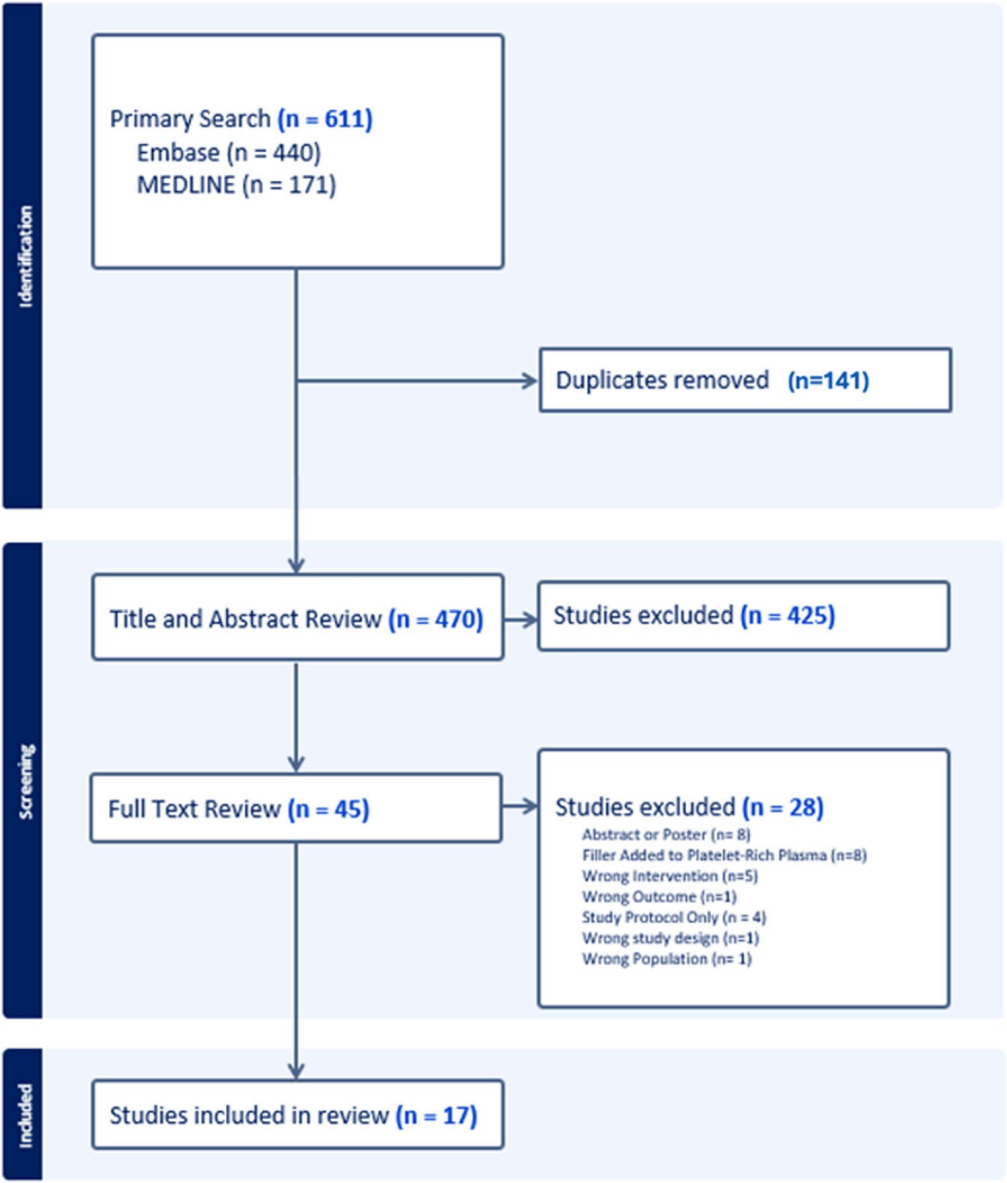
Flowchart for study selection

The primary literature search was carried out until September 2025 and returned 611 articles (MEDLINE *n* = 171, Embase *n* = 440), which after 141 duplicates had been removed left 470 abstracts to screen. Four hundred and twenty-five abstracts were excluded, leaving 45 studies for full text review. Seventeen studies met the inclusion criteria and were included for data extraction (Fig. 1).

Of these 17 studies five (29%) were randomised controlled trials (RCT), four (24%) were randomised non-controlled and six (29%) were non-randomised studies (NRS). Two (12%) studies were retrospective. 11 of 17 studies were split-face studies.

## Data synthesis and assessment of heterogeneity

Substantial heterogeneity was observed across studies in PRP preparation methods, AFCL parameters, and outcome measurement tools. Accordingly, a meta-analysis was not conducted, and results are presented narratively.

### Study characteristics

Seven (41%) studies originated from India, six (35%) studies originated from Egypt and two (12%) from China. Thirteen (76%) studies compared AFCL and PRP against a control group of AFCL alone or AFCL with saline. Other studies compared AFCL and PRP against non-control interventions including stem cell-conditioned medium, topical insulin, PRP gel and microneedling (MN).

Two studies investigated a population with burns or trauma scars, whilst the remaining studies investigated populations with acne scarring. The range of study participants was 12–39. Study characteristics can be seen in Table 1.

**Table 1:** Study Characteristics

Author, Year	Country	Sample size	Scar aetiology	Study design	Study arms	Gender Male: Female	Age Mean +-SD	Treatment protocol	Outcomes measured	Follow up
*Abdel-Maguid* *2021 *[21]	Egypt	16	Acne	Randomised split-face	AFCL + T-PRP vs. AFCL + Stem Cell- conditioned Medium vs. AFCL	7:9	25.88 ± 7.6	3 sessions once monthly	1. Clinical improvement (ECCA score) 2. Patient satisfaction 3. Adverse effects	Monthly and 3 months after last session
*AlTaweel* *2019 *[22]	Egypt	20	Acne	Randomised comparative	AFCL + I-PRP vs.Carboxytherapy + I-PRP	5:15	28.65 ± 7.74	3 sessions once monthly	1. Clinical improvement 2. Patient satisfaction 3. Adverse effects	Monthly and 3 months after last session
*Arsiwala* *2020 *[13]	India	12	Acne	Randomised comparative	AFCL vs. AFCL + T-PRP	12:21*	24.36 ± 4.37*	3 sessions once monthly	1. Clinical improvement (Goodman and Baron quantitative score) 2. Patient VAS score 3. Adverse effects	Monthly for 3 months
*Dai* *2021n *[23]	China	31	Burn hypertrophic scar	Retrospective comparative	AFCL + T-PRP vs. AFCL	19:12	41 ± 12.70	6 sessions once monthly	1. Clinical improvement (VSS) 2. Patient-reported outcome (UNC4P)	Monthly for 7 months
*Galal* *2019 *[24]	Egypt	30	Acne	Randomised split-face	AFCL vs. AFCL + I-PRP	9:21	26.7 ± 4.7	3 sessions once monthly	1. Clinical improvement (Goodman and Baron quantitative score) 2. Patient satisfaction 3. LED camera skin analysis (depth, skin smoothness, scar severity, erythema and pigmentation)	Monthly for 3 months and at 6 months
*Gawdat* *2022 *[25]	Egypt	18	Acne	Randomised split-face	AFCL + I-PRP vs. AFCL + I-PRP gel	3:15	28.33 ± 9.8	3 sessions once monthly	1. Clinical improvement (ECCA score) 2. Patient satisfaction 3. Adverse effects 4. Optical Coherence Tomography	Baseline, one month and 6 months
*Gawdat* *2014 *[26]	Egypt	15	Acne	Randomised comparative split-face	I) AFCL + I-PRP vs. AFCL + Saline II) AFCL + I-PRP vs. AFCL + T-PRP	7:8	24.3 ± 3.7	3 sessions once monthly	1. Clinical improvement 2. Patient satisfaction 3. Adverse effects 4. Optical Coherence Tomography	Monthly for 6 months
*Godara* *2020 *[27]	India	30	Trauma and Burn	Randomised comparative	AFCL vs. AFCL + I-PRP	n/a	n/a	4 sessions once monthly	1. Clinician improvement (POSAS scale) 2. Patient satisfaction (POSAS scale)	Monthly for 5 months
*Guo* *2023 *[28]	China	39	Acne	Retrospective comparative	AFCL vs. AFCL + T-PRP	23:16	24.7 ± 5.8	3 sessions once monthly	1. Clinical improvement 2. Patient satisfaction (Numerical Rating scale, Kolcaba’s general comfort questionnaire, Anxiety and Depression scale, Acne-QOL) 3. Adverse events 4. Gray level value analysis 5. VISIA digital skin analyser	6 monthly for 2 years
*Kar* *2017 *[29]	India	30	Acne	Split-face study (non-randomised)	AFCL vs. AFCL + T-PRP	20:10	25.06 ± 4.44	3 sessions once monthly	1. Clinical improvement (Goodman and Baron qualitative score, visual scar assessment question) 2. Patient satisfaction (visual scar assessment question) 3. Adverse effects	Monthly for 4 months
*Lee* *2011 *[30]	Korea	14	Acne	Split-face study (non-randomised)	AFCL + I-PRP vs. AFCL + saline	10:4	28.1	2 sessions once monthly	1. Clinical improvement 2. Adverse events 3. Chromameter-rated erythema	
*Priya* *2023 *[31]	India	32	Acne	Split-face study (non-randomised)	AFCL vs.AFCL + I-PRP	14:18	n/a	3 sessions six weekly	1. Clinical improvement (Goodman and Baron qualitative and quantitative score, VAS) 2. Patient satisfaction (VAS) 3. Adverse effects	6 weekly for 5 months
*Rageh* *2025 *[32]	Egypt	30	Acne	Randomised split-face	AFCL + T-PRP vs.AFCL + T-insulin	11:19	25.7 ± 4.6	4 sessions once monthly	1. Clinical improvement (Acne Scar Assessment Scale) 2. Patient satisfaction 3. Adverse effects	Baseline and at 5 months
*Sharma* *2025 *[33]	India	30	Acne	Split-face study (non-randomised)	AFCL + I-PRP vs. MN and I-PRP	17:13	28.1 ± 5.4	3 sessions once monthly	1. Clinical improvement (Goodman and Baron qualitative score) 2. Patient satisfaction 3. Adverse effects 4. Global Photographic assessment	Monthly for 3 months
*Sharma* *2021 *[34]	India	30	Acne	Split-face study (non-randomised)	AFCL vs.AFCL + T-PRP	17:13	26.93 ± 4.77	4 sessions once monthly	1. Clinical improvement (Goodman and baron qualitative scale) 2. Patient satisfaction 3. Adverse effects	Baseline and at 6 months
*Solanki* *2020 *[35]	India	26	Acne	Split-face study (non-randomised)	AFCL + saline vs.AFCL + I-PRP	16:10	25.18	6 sessions three weekly	1. Clinical improvement (Goodman and Baron qualitative and quantitative score) 2. Patient satisfaction (10-point score)	Baseline and after treatment
*Ur Rahman* *2024 *[36]	Pakistan	33	Acne	Randomised comparative	MN + I-PRP vs.AFCL + I-PRP	18:15	28.69 ± 6.21*	3 sessions once monthly	1. Clinical improvement (Global acne scarring classification)	Baseline and at 3 months

ECCA, Échelle d'évaluation clinique des cicatrices d'acné; VAS, visual analog scale; VSS, Vancouver scar scale; POSAS, patient observer scar assessment scale; I-PRP, Intradermal PRP; T-PRP, Topical PRP.

* Demographic data only available for entire cohort, as opposed to AFCL+PRP group only

**Study additionally smeared platelet-poor plasma to both sides of the face

### PRP preparation

A double spin method for was implemented in 15 (88%) of studies’ PRP preparation protocols and two (12%) implemented a single spin method [24, 28]. Double spin studies consisted of a first ‘soft’ spin, with mean revolutions per minute 1659 (1000–3000 rpm) for a mean time of 9.3 min (3–15 min), with the second ‘hard’ spin undertaken at a mean of 2939 rpm (1600-4000 rpm) for 10.1 min (2–20 min) (Supplementary Table 1).

Eight (47%) studies administered PRP topically, whilst 10 (59%) studies administered PRP via the intradermal, route with a single study comparing both administration routes. Studies that administered PRP via the intradermal route injected 0.1 ml-0.3 ml per injection site and spaced them 1–2 cm apart, for a total volume of 0.6 ml to 2 ml. Only two of the 10 studies delivering intradermal PRP reported injection volume, injection site spacing and total volume injected.

Intradermal studies that added activators to their PRP formulation were excluded according to the exclusion criteria due to their potential volumetric effect. Three of eight topical studies added an activator prior to administration, either 3% calcium chloride or 10% calcium gluconate (Supplementary Table 1).

### Laser protocol

AFCL settings were invariably reported, as seen in Supplementary Table 2. All studies utilised a CO_2_ laser with a wavelength of 10,600 nm. Pulse energy was 25 mJ − 250 mJ. Laser wattage varied from 10 to 30 W. Dwell time ranged from 0.1ms – 1.54ms. Spacing varied from 0.5 mm to 2 mm. Smart stack level 2 was most used (*n* = 5). Studies most commonly had 3 laser sessions once monthly (*n* = 11). Other studies had two (*n* = 1), four (*n* = 3) or six (*n* = 1) laser treatment sessions.

### Combination therapy versus monotherapy

#### Intradermal PRP

11 studies compared AFCL and PRP against AFCL monotherapy (Table 2). Six of these studies administered PRP topically and five via the intradermal route. The intradermal PRP studies demonstrated an improvement in clinician-reported outcomes in the combination group compared to the monotherapy [24, 30, 31]. This was a statistically significant improvement in two of five intradermal PRP studies. Three of five studies also demonstrated an improvement in pain, erythema and oedema in the combination group of which two were a statistically significant improvement [30, 34]. Two studies reported no difference in adverse effects between groups [27, 31]. Overall, the patient-reported outcomes demonstrated greater satisfaction in the combination groups compared to monotherapy.

**Table 2:** Study Findings

Author, Year	Study design	Study arms	Treatment protocol	Clinical outcomes summary	Patient-reported outcomes summary	Adverse events summary
*Abdel-Maguid* *2021 *[21]	Randomised split-face	AFCL + T-PRP vs. AFCL + Stem Cell- conditioned Medium vs. AFCL	3 sessions once monthly	Significantly greater improvement with AFCL + PRP compared to other intervention (*p* = 0.033)	No significant difference between sides (*p* = 0.412)	No significant difference between sides
*AlTaweel* *2019 *[22]	Randomised comparative	AFCL + I-PRP vs.Carboxytherapy + I-PRP	3 sessions once monthly	Significantly greater improvement with AFCL + PRP compared to other intervention (*p* = 0.039)	No significant difference between groups (*p* = 0.687)	Significantly higher oedema in AFCL + PRP (p value not reported)
*Arsiwala* *2020 *[13]	Randomised comparative	AFCL vs. AFCL + T-PRP	3 sessions once monthly	Greater improvement with AFCL + PRP group compared to control (*p* = 0.129)	Greater satisfaction in AFCL + PRP group (*p* > 0.05)	No significant difference between groups
*Dai* *2021 *[23]	Retrospective comparative	AFCL + T-PRP vs. AFCL	6 sessions once monthly	Significantly greater improvement with AFCL + PRP group compared to control (*p* < 0.05)	NR	Significantly fewer adverse events in AFCL + PRP group (*p* < 0.05)
*Galal* *2019 *[24]	Randomised split-face	AFCL vs. AFCL + I-PRP	3 sessions once monthly	Significantly greater improvement with AFCL + PRP side compared to control (*p* < 0.0001)	Greater satisfaction in AFCL + PRP side (p-values not reported)	Fewer adverse events in AFCL + PRP side (no numerical data reported)
*Gawdat* *2022 *[25]	Randomised split-face	AFCL + I-PRP vs. AFCL + I-PRP gel	3 sessions once monthly	Significant improvement from baseline score for PRP-Gel and PRP-Fluid (*p* = 0.012, *p* = 0.003). No significant difference between sides	Statistical comparison not performed between sides.	Significantly lower pain in AFCL + PRP side (*p* = 0.004)
*Gawdat* *2014 *[26]	Randomised comparative split-face	I) AFCL + I-PRP vs. AFCL + Saline II) AFCL + I-PRP vs. AFCL + T-PRP	3 sessions once monthly	Significantly greater improvement with AFCL + PRP(i) and AFCL + PRP(t) sides compared to control (*p* = 0.03). No significant difference between groups	Greater satisfaction in AFCL + PRP sides vs. control (p-value not reported)	Significantly reduced pain in AFCL + PRP sides vs. intervention (*p* = 0.005) and reduced downtime compared to control (*p* = 0.02)
*Godara* *2020 *[27]	Randomised comparative	AFCL vs. AFCL + I-PRP	4 sessions once monthly	Greater improvement with AFCL + PRP group compared to control (*p* > 0.05)	No significant difference between groups.	No significant difference between groups
*Guo* *2023 *[28]	Retrospective comparative	AFCL vs. AFCL + T-PRP	3 sessions once monthly	Significantly greater improvement with AFCL + PRP group compared to control (*p* = 0.03)	Significantly greater satisfaction in AFCL + PRP group (*p* < 0.05)	Scar scabbing time and decrustation time significantly lower in AFCL + PRP group (*p* < 0.05)
*Kar* *2017 *[29]	Split-face study (non-randomised)	AFCL vs. AFCL + T-PRP	3 sessions once monthly	No significant improvement with AFCL + PRP compared to control (*p* = 0.1242)	Comparison data not collected	Significantly fewer adverse events in AFCL + PRP side (*p* < 0.05)
*Lee* *2011 *[30]	Split-face study (non-randomised)	AFCL + I-PRP vs. AFCL + saline	2 sessions once monthly	Significantly greater improvement with AFCL + PRP side compared to control (*p* = 0.03)	NR	Significantly fewer adverse events in AFCL + PRP side (*p* < 0.05)
*Priya* *2023 *[31]	Split-face study (non-randomised)	AFCL vs.AFCL + I-PRP	3 sessions six weekly	Greater improvement with AFCL + PRP compared to control (*p* = 0.814)	Greater satisfaction in AFCL + PRP side	No significant difference between sides
*Rageh* *2025 *[32]	Randomised split-face	AFCL + T-PRP vs.AFCL + T-insulin	4 sessions once monthly	No significant improvement with AFCL + PRP compared to other intervention (*p* = 0.794)	No significant difference between sides (*p* = 0.276)	Significantly reduced down time in AFCL + PRP side compared to intervention (*p* < 0.001)
*Sharma* *2025 *[33]	Split-face study (non-randomised)	AFCL vs.AFCL + T-PRP	4 sessions once monthly	Significantly greater improvement with AFCL + PRP side compared to control (*p* < 0.05)	Greater satisfaction in AFCL + PRP side (*p* = 0.001)	Significantly fewer adverse events in AFCL + PRP side at first follow-up (*p* = 0.045)
*Sharma* *2021 *[34]	Split-face study (non-randomised)	AFCL + saline vs.AFCL + I-PRP	6 sessions three weekly	Significant improvement with AFCL + PRP from baseline but no statistical comparison was completed between groups	Greater satisfaction in AFCL + PRP side (no p-values reported)	Significantly fewer adverse events in AFCL + PRP side (*p* < 0.05)
*Solanki* *2020 *[35]	Split-face study (non-randomised)	AFCL + I-PRP vs. MN and I-PRP	3 sessions once monthly	Significantly greater improvement with AFCL + PRP side compared to control (*p* < 0.05)	Significantly greater satisfaction in AFCL + PRP group (*p* = 0.0251)	NR
*Ur Rahman* *2024 *[36]	Randomised comparative	MN + I-PRP vs.AFCL + I-PRP	3 sessions once monthly	PRP + MF had significant improvement compared to AFCL + PRP (*p* < 0.05)	NR	NR

*AFCL*, Ablative Fractional Carbon Dioxide Laser; *I-PRP*, Intradermal *PRP*; T-PRP, Topical PRP; *NR*, Not Reported; *POSAS*, Patient observer scar assessment scale; *MN*, Microneedling

### Topical PRP

Among the studies comparing AFCL–PRP combination therapy with AFCL monotherapy, those using topical PRP similarly demonstrated superior outcomes for the combination group. Five of six topically administered PRP studies demonstrated superior clinician-reported outcomes in the combination therapy group compared to the monotherapy group [13, 23, 26, 28, 33]. This was a statistically significant improvement for four studies. There was a significant improvement in patient satisfaction in all six studies. In studies that performed statistical analysis between combination and monotherapy groups, patient satisfaction was significantly higher in the combination group in two of three studies [28, 33]. Adverse events were less common in the combination group in all six studies, and this was statistically significant in four studies.

### Combined therapy versus other interventions

Six studies compared AFCL and PRP to other interventions. These include topical mesenchymal stem-cell condition medium (SCCM), carboxytherapy (intradermal injection of gaseous CO_2_), PRP gel, topical insulin and microneedling. In three of these studies, AFCL and PRP had improved clinician-reported outcomes compared to AFCL and topical SCCM (*p* = 0.006), carboxytherapy (*p*=−0.039) and microneedling and PRP (*p* < 0.05). There was no significant difference in clinical reported outcomes compared to the intervention of AFCL and PRP gel [25] or AFCL and topical insulin [32]. However, there was significantly reduced downtime (*p* < 0.001) and pain (*p* = 0.005) compared to the intervention in these studies respectively. A study by Ur Rahman et al. compared AFCL and PRP to Microneedling and PRP, with the microneedling group demonstrating significantly greater improvement (*p* < 0.005) [36]. Patient satisfaction or adverse events were not reported in this study. Solanki et al. also compared AFCL and PRP to Microneedling and PRP, but in a non-randomised split-face study. They demonstrated AFCL and PRP had significantly superior results (p = 0.0251) [35].

Gawdat et al. published two studies comparing different methods of PRP administration [25, 26]. In 2014 AFCL was compared with topical and intradermal PRP, alongside saline [26]. Photographs at baseline and at the end of study were rated by blinded physicians using a four-point scale for clinical improvement. Topical PRP (T-PRP) demonstrated significantly greater improvement in skin smoothness compared to AFCL with saline. Scar depth, measured by optical coherence tomography, had significantly improved after treatment in AFCL + PRP groups compared to AFCL with saline (*p* = 0.01). Adverse events, including erythema, pain oedema, post-inflammatory hyperpigmentation, was significantly reduced in the AFCL + T-PRP group compared to AFCL alone (*p* = 0.02). In 2022 a second study by the same team examined intradermal PRP compared to intradermal PRP-gel [25]. Both groups demonstrated significant improvement in ECCA scale scores after treatment (PRP *p* = 0.012, PRP-gel = 0.003). Patient satisfaction significantly increased in both PRP and PRP-gel groups (*p* < 0.001). The numerical pain score was significant lower on the PRP side compared to the PRP-gel side (3.22 ± 1.11 vs. 3.78 ± 1.48; *p* = 0.004). Otherwise, there were no significant differences between both sides regarding the pre- and post- treatment satisfaction scores, and the clinical assessment scores after one and three months .

## Discussion

This systematic review comprised 420 patients from 17 studies using AFCL in combination with PRP, administered intradermally or topically, for the treatment of acne, traumatic, and burn scars. The majority of studies demonstrated significant improvement from baseline in clinician and patient related outcomes. Of the 11 studies who performed an analysis between AFCL + PRP to a control, 9 demonstrated an improvement, 6 of which were statistically significant. AFCL combined with PRP demonstrated a significant clinical improvement, matched with higher patient satisfaction and reduced post-treatment downtime when compared with AFCL alone. Quantitative findings from split-face trials, further support these conclusions, demonstrating significant gains in both objective (e.g., scar depth, pigmentation, erythema) and subjective (e.g., global scar scores) measures of scar quality. This highlights the emerging evidence base for AFCL-PRP dual therapy as a viable and effective treatment pathway for scarring in acne, burns and trauma.

Multiple treatment modalities exist for burn and acne scars, either as monotherapy or in combination, yet consensus on the optimal treatment strategy remains lacking. Treating scars with AFCL and PRP poses several advantages: the approach is site-specific, allows for deeper penetration of topical therapies, and offers a less invasive alternative compared with surgical excision or steroid injection, which carry higher recurrence rates and procedural risks. Conservative treatments such as silicone sheeting and compression garments also rely heavily on patient adherence, which can be challenging in clinical practice.

One common adverse event with AFCL is hyperpigmentation, particularly in patients with darker skin phenotypes. Within the studies reviewed, four looked at hyperpigmentation as an adverse event. All studies demonstrated reduced hyperpigmentation in AFCL-PRP versus AFCL monotherapy with two of these a significant difference [24, 34]. Notably, Galal et al. did not report post-inflammatory hyperpigmentation despite 70% of participants having darker skin [24]. Combining PRP with AFCL may mitigate these limitations by accelerating tissue regeneration and reducing downtime-related adverse effects. This is an important consideration when designing treatment protocols and improving patient outcomes for treatment of scarring.

A key challenge within the field is the absence of standardised treatment protocols for AFCL + PRP. No national guidelines currently exist for the combined approach. Although study protocols are working toward evidence-based treatment recommendations, neither addresses the combined use of AFCL and PRP [37, 38]. This highlights an important gap in the literature, with an urgent need for multicentre, adequately powered randomised controlled trials to evaluate this combination therapy.

This review exhibits several limitations, largely related to the quality of evidence, with marked heterogeneity of study protocols and outcome measures, limiting the generalisability of any findings. Significant variability in PRP preparation methods was noted across studies, including differences in centrifuge settings and the quantity applied either topically or via injection. Literature demonstrates that commercial PRP systems yield markedly different concentrations of platelet-derived growth factors and activation levels, potentially influencing treatment outcomes [39–41]. To minimise this source of heterogeneity, studies using exogenous activators during the final preparation step were excluded, as these can alter platelet activity and introduce volumetric effects that confound scar assessment. While AFCL parameters were comparatively more standardised, the number of treatment sessions ranged from two to six, and one study implemented a delayed PRP application protocol, reflecting the challenge of balancing methodological consistency with individualised treatment.

The use of variable clinician-reported and patient-reported outcome measures further complicates generalisability of results and limits the ability to perform robust meta-analyses. This lack of standardisation highlights a fundamental challenge in building a high-quality evidence base for this therapy and presents a clear need for national or international consensus on best practice in research related to scar therapies.

Given the encouraging preliminary results but inconsistent methodology of existing studies, future trials should evaluate AFCL + PRP specifically in acne and burn scars as distinct cohorts. The different aetiologies and healing dynamics of these conditions warrant separate, condition-specific randomised controlled trials to establish reliable treatment algorithms and optimise patient outcomes.

Future research should prioritise the development of standardised, evidence-based protocols for PRP preparation and AFCL settings, as well as uniform outcome measures for scar assessment. Large, multicentre randomised controlled trials with long-term follow-up are essential to validate these protocols and allow direct comparison across studies. Although evidence is still preliminary, AFCL + PRP is emerging as a promising treatment option and could represent a future mainstay of long-term scar and burn scar management. This is particularly significant given the scarcity of emerging scar treatments in this field and the lifelong psychological and physical burden of scarring.

## Supplementary Information

Below is the link to the electronic supplementary material.

Supplementary Material 1 (DOCX 20.7 KB)

Supplementary Material 2 (JPEG146 KB)

Supplementary Material 3 (JPEG161 KB)

## Data Availability

No datasets were generated or analysed during the current study.
