# Supplementary material for: Platelet-rich plasma and ablative fractional carbon dioxide laser therapy for chronic scar management: a systematic review
Source: Lasers Med Sci. 2026 Apr 22;41(1):77. doi: 10.1007/s10103-026-04860-1 (PMC13099715; doi:10.1007/s10103-026-04860-1)
Supplement: Supplementary file 1 — Supplementary Material 1 (DOCX 20.7 KB) [file 10103_2026_4860_MOESM1_ESM.docx]

**Supplementary Table 1**

*PRP Preparation methodology*

*CaCl_2_, Calcium Chloride; rpm, revolutions per minute; Ca, calcium; min, minute; cm, centimetre; n/a, not applicable; cm^2^, centimetre squared.*

| **Author,**  **Year** | **PRP Administration method** | **PRP Spin settings**  (Spin 1 / Spin 2 | **Administration** | **Activator added for topical PRP studies** |
| --- | --- | --- | --- | --- |
| *Abdel-Maguid*  *2021* | Topical* | 160 g for 10 min  400 g for 10 min | NR | 1ml of 3% CaCl_2_ |
| *Al-Taweel*  *2019* | Intradermal | 1500 rpm for 6 min  2500 rpm for 15 min | NR | n/a |
| *Arsiwala*  *2020* | Topical | 1600 rpm for 7 min  4000rpm for 2 min | NR | Not added |
| *Dai*  *2021* | Topical | 2750 rpm for 10 min  2750 rpm for 5 min | NR | Not added |
| *Galal*  *2019* | Intradermal | 1200 g for 6 min | NR | n/a |
| *Gawdat*  *2022* | Intradermal | 1100 rpm for 10 min  2000 rpm for 15 min | Injected 15 sites 1cm apart | n/a |
| *Gawdat*  *2014* | Intradermal and Topical | 150 g for 15 min 400 g for 10 min | 2ml topically applied at 10 different sites 1.5cm apart | 1ml of 3% CaCl2 |
| *Godara*  *2020* | Intradermal | 2316 rpm for 5 min  3538 rpm for 17 min | 0.1ml at 1cm interval | n/a |
| *Guo*  *2023* | Topical | 1400 g for 15 min | 0.5 mm thickness smeared and then reapplied | 10% Ca Gluconate 1:10 ratio |
| *Kar*  *2017* | Topical | 1500 rpm for 10 min  3000 rpm for 20 min | NR | Not added |
| *Lee*  *2011* | Intradermal | 3000 rpm for 3 min  4000 rpm for 3 min | 0.3ml at 1.5-2cm intervals at 20 sites | n/a |
| *Priya*  *2023* | Intradermal | 1000 rpm for 10 min  2000 rpm for 5 min | NR | n/a |
| *Rageh*  *2025* | Topical | 1500 rpm for 10 min  4000 rpm for 5 min | NR | Not added |
| *Sharma*  *2025* | Topical | 1000 rpm for 10 min  2000rpm for 5 min | NR | Not added |
| *Sharma*  *2021* | Intradermal | 1500 rpm for 15 min  3000 rpm for 15 min | 0.2ml at 2cm interval up to 1-2ml | n/a |
| *Solanki*  *2020* | Intradermal | 1300 rpm for 15 min  1600 rpm for 15 min | 0.01 ml per cm^2^ | n/a |
| *Rahman*  *2024* | Intradermal | 1500 rpm for 10 min  4000 rpm for 10 min | NR | n/a |

**Supplementary Table 2**

*Laser settings*

*mJ, millijoules; mm, millimetres; W, watts; NR, not reported; Hz, hertz;* *cm^2^, centimetre squared.*

| **Author,**  **Year** | **Pulse energy, density and number of passes** | **Power (wattage)** | **Dwell time** | **Spacing** | **Level** |
| --- | --- | --- | --- | --- | --- |
| *Abdel-Maguid*  *2021* | 42-45mJ  Spot density 100/cm^2^ | NR | NR | NR | 1-2 |
| *AlTaweel*  *2019* | NR | 15W | 600 ms | 0.7 mm | 3 |
| *Arsiwala*  *2020* | 50-100mJ  Spot density 50-100/cm^2^  1-2 passes | 30W | NR | NR | NR |
| *Dai*  *2021* | 17.5-100mJ  density of 3–5% and a frequency of 250 Hz | NR | NR | NR | NR |
| *Galal*  *2019* | NR | 15W | 0.6 ms | 0.8 mm | 2 |
| *Gawdat*  *2022* | NR | 18W | 0.5 ms | 0.5 mm | 2 |
| *Gawdat*  *2014* | NR | 15W | 0.6 ms | 0.7 mm | 2 |
| *Godara*  *2020* | 25mJ  Spot density 144/cm^2^ | NR | 0.1 ms | 0.5mm | NR |
| *Guo 2023* | 42-60mJ  10-25% coverage rate | NR | NR | 1-2 mm | NR |
| *Kar*  *2017* | 200-250mJ | 30W | 1.54 ms | 0.8 mm | 6-8 |
| *Lee*  *2011* | 25mJ per 150 μm diameter  Density of 400 MTZ/cm^2^ | NR | NR | NR | NR |
| *Priya*  *2023* | 1-2 passes | 15W | 0.5 ms | 0.5mm | NR |
| *Rageh*  *2025* | 1 pass | 15W | 0.6 ms | 0.8 mm | 2 |
| *Sharma*  *2025* | NR | 10-30W | 1 ms | 1 mm | NR |
| *Sharma*  *2021* | 25–45 mJ/cm^2^  1 pass | NR | NR | NR | NR |
| *Solanki*  *2020* | 15mJ | NR | NR | NR | 1 |
| *UrRahman*  *2024* | NR | NR | NR | NR | NR |
