# Supplementary figures and images for: Platelet-rich plasma and ablative fractional carbon dioxide laser therapy for chronic scar management: a systematic review
Source: Lasers Med Sci. 2026 Apr 22;41(1):77. doi: 10.1007/s10103-026-04860-1 (PMC13099715; doi:10.1007/s10103-026-04860-1)

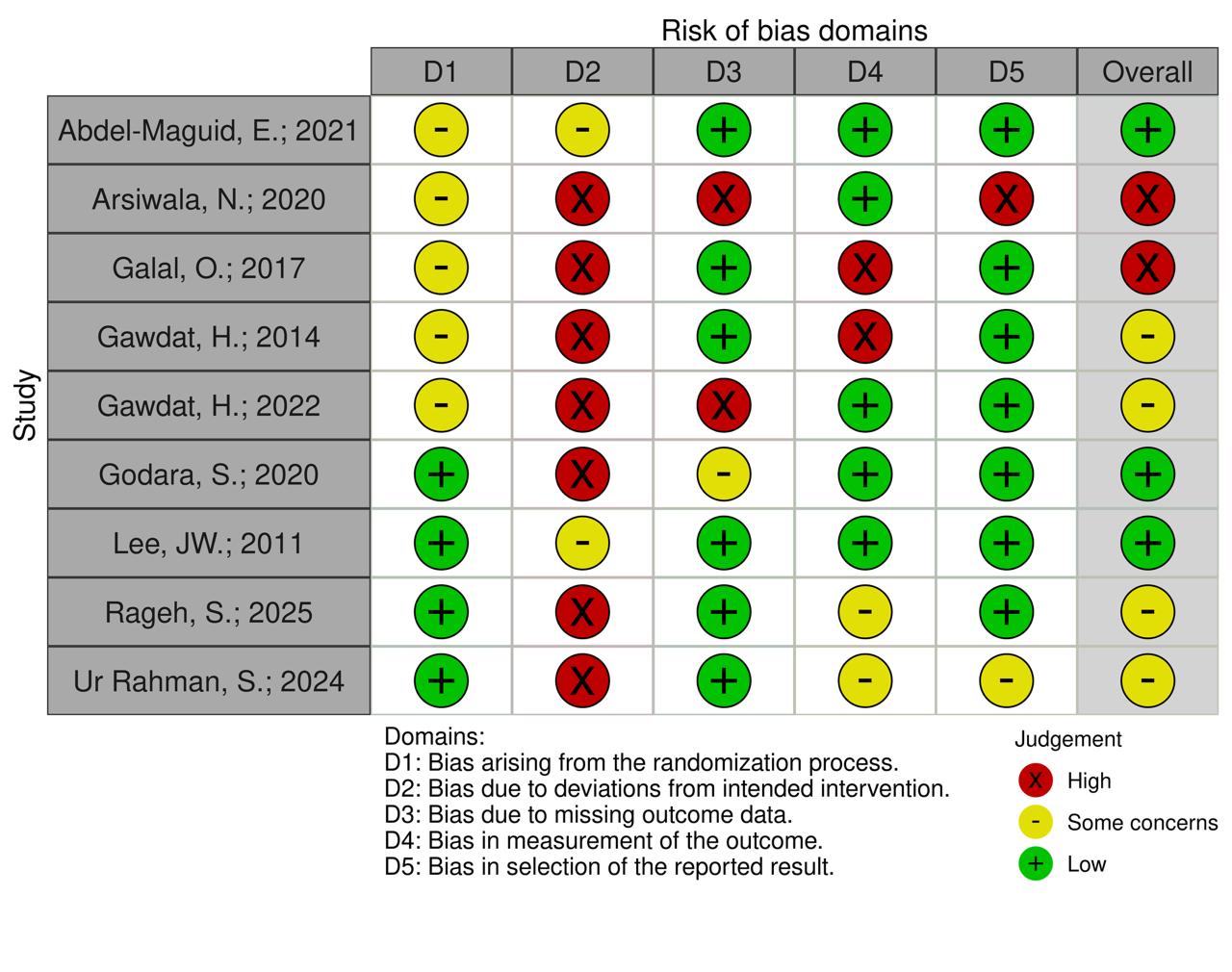

Supplement: Supplementary file 2 — Supplementary Material 2 (JPEG146 KB) [file 10103_2026_4860_MOESM2_ESM.jpeg]

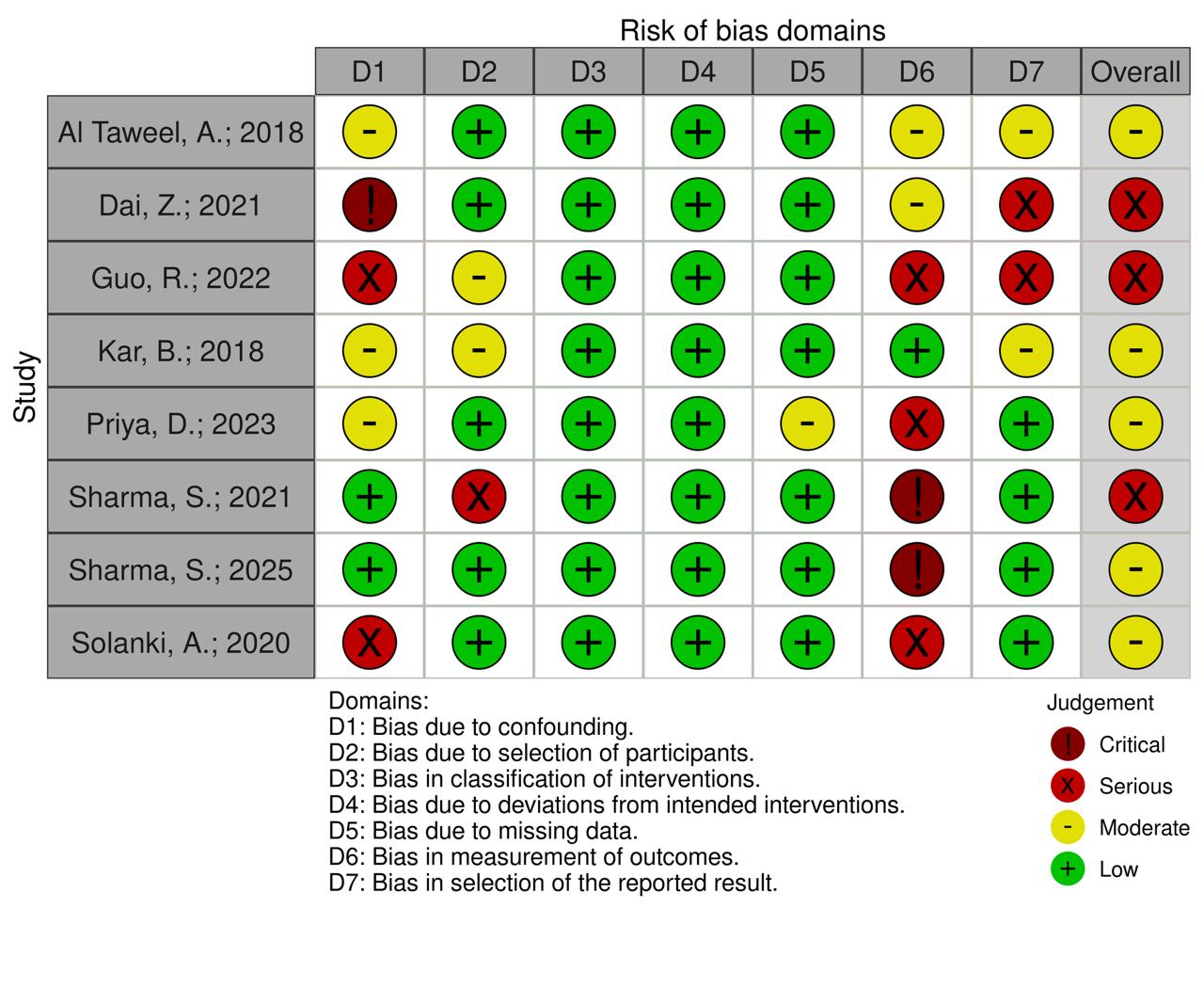

Supplement: Supplementary file 3 — Supplementary Material 3 (JPEG161 KB) [file 10103_2026_4860_MOESM3_ESM.jpeg]
